# Larger than Life: Humans' Nonverbal Status Cues Alter Perceived Size

**DOI:** 10.1371/journal.pone.0005707

**Published:** 2009-05-27

**Authors:** Abigail A. Marsh, Henry H. Yu, Julia C. Schechter, R. J. R. Blair

**Affiliations:** 1 Department of Psychology, Georgetown University, Washington, D. C., United States of America; 2 Mood & Anxiety Program, National Institute of Mental Health, Bethesda, Maryland, United States of America; Indiana University, United States of America

## Abstract

**Background:**

Social dominance and physical size are closely linked. Nonverbal dominance displays in many non-human species are known to increase the displayer's apparent size. Humans also employ a variety of nonverbal cues that increase apparent status, but it is not yet known whether these cues function via a similar mechanism: by increasing the displayer's apparent size.

**Methodology/Principal Finding:**

We generated stimuli in which actors displayed high status, neutral, or low status cues that were drawn from the findings of a recent meta-analysis. We then conducted four studies that indicated that nonverbal cues that increase apparent status do so by increasing the perceived size of the displayer. Experiment 1 demonstrated that nonverbal status cues affect perceivers' judgments of physical size. The results of Experiment 2 showed that altering simple perceptual cues can affect judgments of both size and perceived status. Experiment 3 used objective measurements to demonstrate that status cues change targets' apparent size in the two-dimensional plane visible to a perceiver, and Experiment 4 showed that changes in perceived size mediate changes in perceived status, and that the cue most associated with this phenomenon is postural openness.

**Conclusions/Significance:**

We conclude that nonverbal cues associated with social dominance also affect the perceived size of the displayer. This suggests that certain nonverbal dominance cues in humans may function as they do in other species: by creating the appearance of changes in physical size.

## Introduction

Social dominance and physical size are inextricably linked. In species ranging from montane lizards [1] to mountain gorillas [2], physical size is a direct and primary determinant of social dominance, with physically larger animals attaining greater status than smaller animals [3], [4]. Physical size is associated with maturity and strength and allows larger animals to prevail in physical competitions [5]. It is therefore not surprising that many species' behavioral dominance cues cause the animal to *appear* physically larger [6], [7]. Appearing larger may enhance social dominance because larger-appearing opponents are more likely to spur an opponent to withdraw and thus win by forfeiture. It has not yet been tested whether humans' nonverbal dominance cues function in a similar way: by altering perceived size. The results of four studies we conducted show that high status and low status cues lead to changes in apparent physical size, and the extent to which nonverbal status cues such as body posture alter apparent size predicts how effective they will be in conveying social dominance. These results suggest that the nonverbal dominance cues used by humans and other animals serve parallel functions.

Social dominance facilitates success in competition for territory, reproduction, and survival in many species. Greater physical size enhances non-human animals' ability to attain these goals [4]. In humans, physical size also confers advantages in social dominance and the acquisition of resources. Taller men earn more money (as much as $600 per inch) [8]–[10] and achieve higher job status [9], [11]. Ten of the twelve United States presidential elections from 1952 to 1996 were won by the taller candidate [12]. Accordingly, several psychological studies have demonstrated that physical size affects perceptions of status [13], [14] and that status alters perceptions of physical size [15]–[17]. However, no prior study has assessed whether human nonverbal cues can, like the nonverbal cues of non-human animals, create the appearance of changes in physical size that influence the displayer's perceived status. The present study was conducted to address this question.

What could cause size to be misperceived as a function of perceived status? One mechanism, as suggested by Higham and Carment [15] and by Wilson [17], is that perceivers infer that high status targets are larger than low status targets. Another possible mechanism is that cues shown by high status people could cause them to literally appear larger. A recent meta-analysis described hierarchy cues that most reliably lead to changes in perceived social status [18]. A wide variety of nonverbal cues can lead to changes in apparent status, including nodding, shifting the legs and body, raising and lowering the brows, and maintaining greater or lesser interpersonal distance. Several cues that are particularly closely associated with status are those that could change perceptions of apparent size. These include postural openness, outwardly directed hand or arm gestures, facing orientation, and reduced interpersonal distance. Of these, postural openness has been seen to characterize higher status individuals' actual behavior in natural or laboratory settings [18]. An open posture, for example, is more likely to be observed in the winner of an athletic competition than in the loser [19]. One possibility is that cues like this alter the displayer's perceived size, thereby influencing perceivers' status attributions.

The display of dominance cues that enhance apparent physical size is common among many non-human animals. Actual physical size confers costs such as greater energy requirements, which is one reason that species do not continue to expand indefinitely in size [20]. To acquire the benefits of increased size an animal can employ physiological and behavioral changes to simply appear larger, thus improving its chances of winning status competitions [21]. Animals seeking to become dominant may seem to “grow in size” (p. 62) [22], and dominant animals stand taller than subordinates [23]. During competitive or aggressive encounters, fish may engage in behaviors such as broadside displays or raising their dorsal fins, and mammals may exhibit piloerection in which the hair along the spine is raised. These displays increase the size of the silhouette in the two-dimension plane that is visible to perceivers [24], [25]. Other displays in dominance competitions, such as lizards' pushups, increase apparent vertical height [26]. By contrast, low status cues may make an individual appear smaller in size [27]. Submission cues thereby suggest helplessness and weakness to convey a lack of threat. In social animals, the display of submission cues will ordinarily end an attack. In humans, formalized versions of such cues include kneeling or bowing [6], [27].

Some evidence suggests that changes in apparent physical size affect perceived dominance. For example, increasing one's physical elevation by standing on a platform or riser increases apparent status [28]. However, to date no research has assessed whether human nonverbal dominance cues serve a purpose parallel to that of non-human animals' dominance cues: to alter perceptions of dominance by creating the appearance of changes in size. We conducted four studies that demonstrated that the appearance of changes in size also affects perceptions of status. The results confirmed that individuals showing high status nonverbal cues, particularly postural openness, were judged to appear larger than individuals showing lower-status cues, and that these cues' effects on perceived size predicted their effects on perceived dominance.

## Materials and Methods

### Stimuli

The creation and validation of the stimulus set used in all of the following 4 experiments have been described in detail and sample stimuli have been depicted previously [29]. In summary, sixteen actors (8 females; *M* age = 32.5 years, *SD* = 8.91) were recruited via a flier sent to local community theater groups in the Washington, D.C., metropolitan area. The high status and low status poses were composed of cues shown to be highly indicative of perceived dominance and subordination [18]. These cues were brow position, gaze direction, body posture, and gestures. These cues were combined to create 8 high status poses and 8 low status poses. Each actor was also photographed in 8 neutral poses in which neither high or low status cues were present. Half of the poses of each type were seated and half were standing, such that pose type was crossed with the two-level seated/standing variable.

High status variants of the cues were: lowered brows, direct gaze, open body posture, and outwardly-directed gestures, such as pointing. Low status variants of these cues were raised brows, averted gaze, closed posture, and self-directed gestures, such as touching one's own neck. Each of the high status and low status poses combined 3 of the 4 possible cues. For example, one high status seated pose and one high status standing pose shown by each target incorporated high status gaze, brows, and gestures but neutral posture. Another incorporated high status posture, gaze, and gestures, but neutral brows.

In all neutral poses, neutral versions of each of the four status cues were employed: targets' brows were in the neutral position rather than being raised or lowered, they gazed past the camera rather than directly at it or perpendicular to it, their posture was neither opened nor closed, and no self-directed or outwardly directed gestures were employed. Variation was introduced by including in each neutral pose a nonverbal behavior not shown to be relevant to status, such as standing with the weight shifted onto one foot, standing with hands in pockets, or sitting with hands resting on knees.

All of the photographs were taken with a Sony™ digital camera by a single experimenter. The camera was mounted on a tripod in a large room against a white wall. The camera was positioned the same distance away from the actors for all poses and the same chair was used for all of the seated poses. After the photos were collected, they were digitally cropped and converted to grayscale, and any glare in the actors' eyes resulting from the camera flash was corrected using Adobe Photoshop™.

### Experiment 1: High status nonverbal cues increase apparent height

This study was conducted to assess whether nonverbal status cues affect not only attributions of dominance but attributions of physical height and weight as well.

#### Participants

Twenty participants (13 females; *M* age = 29.8 years, *SD* = 8.07) judged targets' physical attributes: their apparent height in inches, weight in pounds, and age in years. All participants enrolled in this and the following studies were recruited in the Washington, D.C., metropolitan area through posted advertisements for behavioral studies. All were screened by a staff physician in the National Institute of Mental Health outpatient clinic at the NIH Clinical Research Center to ensure that they were physically healthy and had no personal history of mood or anxiety disorder, psychosis, or alcohol or drug abuse.

#### Ethics Statement

This research was approved by the Combined Neuroscience Institutional Review Board at the National Institute of Mental Health, and all participants' written informed consent was obtained prior to the study's commencement.

#### Task

Six separate versions of the questionnaire were created, each showing all actors only once, and each participant completed only one of the six questionnaires. This permitted each target to be judged in each type of pose (seated and standing versions of high status, neutral, and low status poses) but each participant to see and judge each target only once. Each of the 4 different variations of each pose type was represented in the six versions of the questionnaire. Participants judged all targets on one attribute before moving on to the next attribute. The order in which the attributes were judged was randomized across participants.

#### Results

Data were analyzed using the targets as the units of analysis to control for variation in targets' actual height (preliminary testing confirmed that targets' self-reported height was associated with perceived dominance). The effective reliability of judgments across seated and standing poses was high (*R* = .96) and so these judgments were collapsed. We conducted a 2 (gender)×3 (low, neutral, high status) ANOVA for which status constituted a repeated-measured variable. The results showed that pose type significantly affected judgments of targets' height, *F*(2, 28) = 7.44, *p*<.005, *η* ^2^ = .35 (Table 1). Targets appeared physically taller in high status and neutral poses than low status poses. Binomial distribution tests showed that differences between high status and low status poses were significant, *p*<.01, as were those between low status and neutral poses, *p*<.05. Differences between high status and neutral poses were not statistically significant, *p*<.07. *T*-tests yielded similar results (respectively, *t*(15) = 3.28, *p*<.005, *r* = .65; *t*(15) = 2.48, *p*<.05, *r* = .54; and *t*(15) = 1.67, *p* = .12, *r* = .40). A main effect of target gender showed that men were judged to appear taller than women, *F*(1, 14) = 26.50, *p*<.001, but no interaction between gender and status cues emerged, *F*(2, 28) = 0.44, *ns*.

**Table 1:** Perceived size as a function of status cues in Experiment 1.

	High status	Neutral	Low status	*p*
Height (inches)	68.8^a^	68.5^a^	67.9^b^	<.005
Weight (pounds)	161.22^a^	160.50^a^	156.44^b^	<.07

Where row notations (a, b) differ indicates significant differences among groups.

A marginally significant effect of status poses on judgments of weight emerged, *F*(2, 28) = 2.96, *p* = .07, *η* ^2^ = .17 (Table 1). Targets appeared physically heavier in high status poses and neutral poses than in low status poses. A binomial distribution test indicated a significant difference between high status and low status poses, *p*<.05 [*t*(15) = 2.13, *p*<.05, *r* = .48]. No significant effect of status cues on perceived age were observed (*p*s>.50).

#### Discussion

This study demonstrated that status cues influence perceptions of physical size, particularly height. However, changes in perceived height might result purely from inferences about higher status individuals being larger [15], [17]. Experiments 2 and 3 were conducted to address whether low-level perceptual processes might also drive the effect of status cues on perceptions of size. Experiment 2 was conducted to establish that manipulating perceived size influences perceived status as well. Digital images of targets showing neutral cues were altered to create the appearance of changes in physical size to assess whether illusory changes in physical size alter naïve perceivers' impressions of social dominance.

### Experiment 2: Altering apparent size by manipulating environmental cues influences perceived status

#### Participants

Thirty-nine participants (31 females; *M* age = 30.4 years, *SD* = 10.2) judged targets' physical attributes: their apparent height in inches, weight in pounds, and age in years, and dominance (1–7 scale). Participants judged all targets on one attribute before moving on to the next attribute. The order in which the attributes were judged was randomized across participants.

#### Task

**Figure 1:**
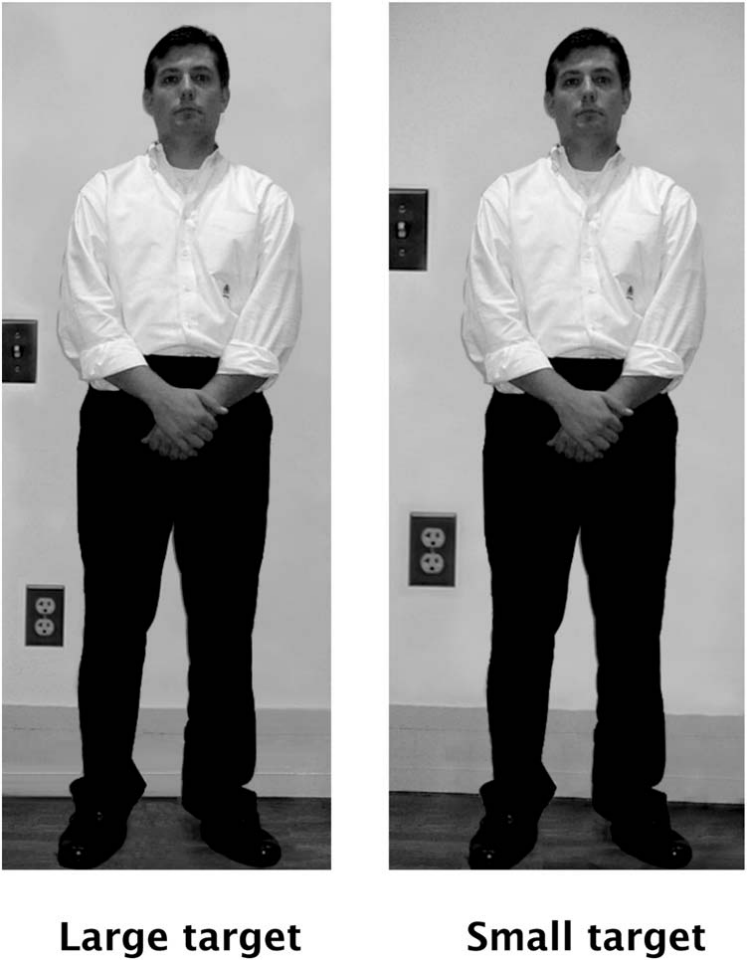
Example of photographs altered to influence perceived size of target.

Each participant completed one of two separate versions of the questionnaire, both of which showed each actor only once. In each version, 8 of the photographs had been manipulated to make the target appear smaller, and 8 to make the target appear larger. Separating the targets into two questionnaires permitted each target to be judged in each type of manipulation, but allowed each participant to see and judge each target only once and to judge targets made to appear both smaller and larger. These “Small Target” and “Large Target” images were identical across conditions, but the size and placement were of an electrical outlet and a light switch panel on the wall behind the target had been manipulated (Figure 1). In the Small Target condition, the light switch measured 27 mm high and was superimposed 167 mm from the floor. The outlet measured 25 mm high and was superimposed 66 mm from the floor. The target thus looked smaller relative to these contextual cues. In the Large Target condition, the light switch measured 21 mm high and was superimposed 131 mm from the floor. The outlet measured 21 mm high and was superimposed 46 mm from the floor. The target thus looked larger relative to these contextual cues. No participant indicated awareness of the digital manipulation when queried following testing.

#### Results

Data were analyzed using the targets as the units of analysis. Binomial distribution comparisons, which are non-parametric tests similar to *t*-tests, indicated that the manipulation affected participants' judgments of both height and dominance. Targets were rated to appear taller in the Large Target condition than the Small Target condition, *p*<.001 (Table 2). Targets were also judged to appear more dominant in the Large Target condition than the Small Target condition, *p*<.05. Parametric *t*-tests similarly indicated that manipulating environmental features to increase apparent size increased participants' estimates of height, *t*(15) = 5.06, *p*<.001, and dominance, *t*(15) = 1.93, *p* = .07, two-tailed. Neither judgments of weight or age were affected by the manipulation (All *p*s>.10).

**Table 2:** Perceived size and status as a function of manipulations of environmental cues in Experiment 2.

	Large	Small	*p*
Height (inches)	68.53	67.16	<.001
Dominance (1–7 scale)	4.37	4.19	<.05

#### Discussion

This study demonstrated that altering perceptual cues to create the appearance of increased size affects perceived dominance. Targets for whom the background had been manipulated to increase their apparent size were judged to appear both taller and more dominant than targets made to appear smaller. Experiments 3 and 4 were conducted to assess whether nonverbal status cues perform a similar function: creating the appearance of increased size, thereby enhancing the perceived social dominance of the expresser.

### Experiment 3: Status cues enlarge objectively measured height and silhouette

#### Methods

This study was conducted to objectively measure whether status cues serve to increase a target's apparent size. In this study, the image size of the target individuals in the stimulus set (which was the stimulus set used for all 4 studies) who were photographed in seated and standing low, neutral, and high status poses, was measured using the histogram function of Adobe Photoshop™. Using this program, the height and width of each target in pixels and the area occupied by each target's silhouette in pixels was measured, generating three values for each of the 24 poses shown by all 16 targets. Measurements of area included enclosed negative space, meaning space entirely visually enclosed by the target's body, such as the space inside a target's arm when his hand is on his hip. These measurements were analyzed using SPSS to investigate whether apparent size in the two-dimensional plane that is visible to a perceiver varied across low, neutral, and high status cues.

#### Results

Three 2 (seated, standing)×2 (gender)×3 (low, neutral, high status) repeated-measures ANOVAs were conducted using the targets themselves as the units of analysis. The dependent variables for the three ANOVAs were, respectively, the targets' height in pixels, width in pixels, and the area in pixels occupied by the targets' silhouette, including negative space. Results confirmed that participants' nonverbal dominance cues significantly affected measured height, *F*(2, 28) = 14.88, *p*<.001, *η* ^2^ = .52 (Table 3). Targets measured taller in high status and neutral poses than low status poses. Paired-samples *t*-tests showed that differences between high status and low status poses were statistically significant, *t*(15) = 4.69, *p*<.001, as were those between low status and neutral poses, *t*(15) = 3.40, *p*<.005, poses. Differences between high status and neutral poses were not significant (*p*>.10). Both gender and whether the pose was seated or standing significantly altered measured height (*p*s<.001), but neither factor interacted with dominance pose (*p*s>.10).

**Table 3:** Measured size as a function of status cues in Experiment 3.

	High status	Neutral	Low status	*p*
Height (pixels)	754.16^a^	750.84^a^	724.02^b^	<.001
Width (pixels)	376.04^a^	265.70^b^	235.36^c^	<.001
Area (pixels)	122,388^a^	121,034^a^	110,725^b^	<.001

Where row notations (a, b) differ indicates significant differences among groups.

Similar results were found for target width and the area occupied by targets' silhouettes. Nonverbal dominance cues significantly affected the width of the targets, *F*(2, 28) = 433.00, p<.001, *η* ^2^ = .97. Targets appeared wider in high status than neutral poses, *t*(15) = 14.64, *p*<.001, and wider in neutral poses than low status poses, *t*(15) = 7.99, *p*<.001, poses. No main effect of interactions for whether the target was seated or standing were observed (*p*s>.10), but a main effect of gender was found (*p*<.001) as well as a pose×gender interaction (*p*<.01), such that the difference in width between men and women was greater for low status and neutral than high status poses.

Finally, nonverbal dominance cues also affected the area occupied by targets' silhouettes, *F*(2, 28) = 17.79, *p*<.001, *η* ^2^ = .56 (Table 3). Targets appeared larger in high and neutral status than low status poses. Low status poses had significantly smaller areas than high status, *t*(15) = 4.63, *p*<.001, and neutral, *t*(15) = 8.31, *p*<.001, poses, but the increase for high status over neutral status poses was not significant (*p*>.10). Both gender and whether the pose was seated or standing significantly altered the area of the measured silhouette (*p*s<.001), but neither variable interacted with dominance pose (*p*s>.10).

#### Discussion

This study confirmed that cues demonstrated to increase perceived status also increase a participant's size as measured using objective determinations of height, width and the area of the silhouette. Although the targets' actual size did not vary across poses, in the sense that their actual height and weight were unchanged, the targets' *apparent* size in the two-dimensional plane visible to a perceiver varied significantly. Notably, the results of this study reflected the influence of only three types of cues that influence the target's silhouette: posture, gesture, and whether the target was seated or standing. This suggests that one or more of these cues are the specific nonverbal behaviors that are responsible for the changes in perceived status that are effected by changes in perceived size. Experiment 4 was conducted to assess three things: 1) to confirm that naïve perceivers detected the changes in apparent size caused by these cues, 2) to assess the relative contribution of the various status cues to this phenomenon, and 3) to assess whether the changes in perceived size mediate changes in perceived status.

### Experiment 4: Effects of specific cues on perceptions of size and dominance

#### Participants

Twenty participants (13 females; *M* age = 27.7 years, *SD* = 6.41) viewed all pictures in the stimulus set and assessed targets' apparent size and dominance.

#### Task

Both size and dominance were judged on a seven-point Likert scale anchored by extreme judgments (e.g., “Very small,” “Very large”; “Very dominant,” “Very submissive”) to make the measures more comparable and test mediation effects.

#### Results

It will be recalled for each status level (high, neutral, low) each actor performed both seated and standing variants of four poses. In order to assess the relative importance of the cues composing the poses, in these analyses we used the 24 poses themselves as the units of analysis. We first conducted a 2 (seated, standing)×3 (low, neutral, high status) ANOVA to confirm that the pose type affected actors' perceived size. The results once again showed a main effect of status level on perceived size, *F*(2, 18) = 72.29, *p*<.001, *η* ^2^ = .89 (Table 4). Targets were judged to appear larger in high status than neutral or low status poses. Low status poses were judged to appear significantly smaller than neutral, poses, *t*(14) = 5.91, *p*<.001, *r* = .84, and neutral poses were judged to appear significantly smaller than high status poses, *t*(14) = 4.23, *p*<.001, *r* = .75. The ANOVA results also indicated that whether the pose was shown seated or standing affected apparent size, *F*(1, 18) = 12.40, *p*<.005, *η* ^2^ = .41. Actors were judged to look larger when standing (*M* = 4.24, *SD* = 0.19) than seated (*M* = 4.16, *SD* = 0.13).

**Table 4:** Perceived size as a function of status cues in Experiment 4.

	High status	Neutral	Low status	*p*
Size (1–7 scale)	4.38^a^	4.20^b^	4.02^c^	<.001

Where row notations (a, b, c) differ indicates significant differences among groups.

We next assessed how each of the cues that composed the poses affected perceived size. Each pose was coded to denote whether the high status (+1) neutral (0) or low status (−1) variant of each type of cue (e.g., postural openness) was present. In addition, we coded each pose as seated or standing. We then conducted a simultaneous multiple regression analyses to determine which of the five types of cues (brows, gaze, gestures, posture, or seated v. standing position) was most significantly associated with size judgments. The results indicated that posture (open versus closed) was most strongly associated with apparent size, followed by the seated v. standing variable. No other cues significantly affected perceived size (Table 5).

**Table 5:** Relative role of status cues in affecting perceived size in Experiment 4.

	*Beta*	*t*	*p*
Postural openness	0.521	4.66	<.001
Seated/standing	0.263	3.59	<.005
Gestures	0.198	1.77	<.10
Gaze	0.171	1.53	<.20
Brows	0.145	1.29	<.30

Overall model: *F*(5, 18) = 33.70, *p*<.001, adjusted *R* ^2^ = .88.

The results of a Sobel test indicated that perceptions of size significantly mediated the relationship between the presence of status-relevant posture cues and perceptions of dominance, *t* = 3.32, *p*<.001. Moreover, posture failed to remain a significant predictor of dominance after the inclusion of perceived size into a second multiple regression analysis (Table 6).

**Table 6:** Relationship between posture and perceived status after accounting for perceived size in Experiment 4.

Model 1	*Beta*	*t*	*p*
Postural openness	0.825	6.85	<.001

Model 2	*Beta*	*t*	*p*
Postural openness	0.223	1.16	>.10
Size	0.70	3.64	<.005

Overall Model 1: *F*(1, 22) = 46.86, *p*<.001, adjusted *R* ^2^ = .67.

Overall Model 2: *F*(2, 21) = 43.06, *p*<.001, adjusted *R* ^2^ = .79.

## Discussion

The results of the preceding studies consistently show that nonverbal status cues influence apparent size. This study is the first that we are aware of to demonstrate that humans' status cues, particularly postural openness, make the displayer appear physically larger and that this appearance mediates perceptions of status. These findings link the function of humans' nonverbal status cues with those of many non-human animals. Experiment 1 showed that status cues affect perceivers' estimations of targets' height in inches and weight in pounds. Experiment 2 confirmed that the relationship between perceived size and status could be due to low-level perceptual processes. The results of Experiments 3 and 4 suggested that nonverbal status cues may also constitute perceptual cues that alter perceived size. Actors showing high status cues, particularly open posture cues, were measured as taller and as presenting actually larger silhouettes to the viewer, and were judged by naïve perceivers to appear physically larger and more dominant than actors showing low status cues. The extent to which perceived size was affected mediated the perceived dominance of the actor. Together, these data suggest that altering perceived size may be an important means by which nonverbal cues such as postural changes create the appearance of social dominance. Postural openness is one of the few nonverbal cues that has been demonstrated to be actually used disproportionately by high status individuals [18]. The present study suggests that this may be the case because it effectively serves to alter status perceptions by changing the displayer's perceived physical size.

How do nonverbal status cues affect viewers' judgments of size? Some have suggested [15], [17] that the process is inferential: the knowledge that height and status are associated leads perceivers to infer that higher status individuals are taller. But this cannot be the entire story, given the data reported in Experiment 3. The results of this study suggest that status cues change the amount of visual space that a target's body occupies. This may then lead observers to misperceive the target's actual size, for example, his or her height. A man who is judged to be 6′0″ when showing high status cues might be judged to be 5′11″ when showing low status cues, although the man's actual height in inches remains unchanged. The results of Experiment 3 suggest that perceivers' judgments of height are associated with objective measurements of image size, although objective measurements are not the only factor that affects height judgments. There were considerable differences in the objectively measured height of targets who stood versus sat. But whether targets stood or sat did not affect perceivers' judgments of height in inches (*p*s>.50) in Experiment 1, whereas the type of status pose displayed did affect these judgments. This pattern of results thus supports the idea that one purpose of status cues is to mislead viewers into misperceiving the target's actual body size.

Common English colloquialisms attest to widely held beliefs about the relationship between height and social status: We “look up” to higher status people, who may be characterized as “elevated,” having reached the “height of power,” “standing head and shoulders” above their peers, or simply being “giants” among them. These beliefs are based, to an extent, on reality. Taller individuals are more likely to hold leadership positions in the workplace [9], earn higher incomes [8], and attract members of the opposite sex (this is particularly true for men) [30]. Height may result in advantageous social outcomes in part because physical size is an indicator of an individual's fitness. Height is correlated with health [31], physical strength [32], longevity [33], and intelligence [34]. Because increased size also confers risks such as increased visibility to predators and the need for required resources, a tall individual demonstrates that he or she has strong enough genes to support his or her extra size. We speculate that high status cues in humans such as an open postural stance may have evolved to create the appearance of larger physical size, thereby helping the expresser to reap the benefits the appearance of greater size confers.

The importance of an expanded posture to perceptions of dominance is well established [18], [35]. In a variety of other species, posture cues are used to influence the outcomes of status competitions. Animals whose flanks provide their largest silhouette will stand sideways to an opponent; other animals will increase their apparent size via piloerection or simply standing up taller [24]–[27]. By simulating a larger appearance, high status cues increase the likelihood of the expresser being perceived as dominant, thereby increasing the chances of eliciting submission from competitors.

Conversely, simulating a smaller appearance is a means of appeasement that may inhibit attack in aggressors. In the present studies, low status cues made targets appear physically smaller to the same or greater degree as high status cues made them appear physically larger. Given the advantages conferred by size, it may seem surprising that cues would be used that reliably make a target appear physically smaller. However, the appearance of reduced size can also confer advantages in competitive or aggressive encounters. These encounters are highly ritualized in many species to prevent serious injuries from ensuing. As observed by Konrad Lorenz [6], in many species submissive behavior involves crouching, lowering the body, or rolling over. This creates an appearance of defenselessness that may be a powerful inhibitor of further aggression [7]. Universally recognized high and low status cues share some overlapping traits with human displays of pride and shame, which are demonstrated after victory or defeat, respectively [36]. The similarity in the types of dominance-related postural changes seen across species suggests a high degree of evolutionary continuity in the use of cues that alter apparent physical size during status displays.

Perceptions of dominance may also be affected by a variety of factors for which the studies described here attempted to control. The appearance of looming or approaching the viewer can create the appearance of threat, which could alter perceived dominance [37]. To control for this, we counterbalanced whether actors were leaning slightly forward or backward across high, neutral, and low status poses. Also, in the manipulated photographs used for Experiment 2, the floorboards and wood strip adjoining the wall remained visible in order to visually anchor the target to the wall and prevent the appearance of looming. Aspects of open posture such as the appearance of relaxation or territorial control could create an appearance of dominance that is independent of changes in size. However, the objective measurements of physical size generated for Experiment 3 support the actual size changes that these postural changes effect. In addition, the mediation effects assessed in Experiment 4 indicate that changes in perceived size are critical to the effectiveness of the postural openness cue.

The present studies did not find gender to significantly moderate the size-dominance relationship, although, as a general rule, gender is an important moderator of status perceptions. Men are often perceived to be higher status than women [38], [39], women and men may use partially distinct dominance cues in their social interactions [40], and the way the status cues of men and women are processed are partially distinct as well [29], [41]. However, in many contexts the status cues that men and women employ are highly similar [35], [38]. This is not surprising, as attaining high status is advantageous for males and females across species [42], [43]. This may help to explain why gender did not significantly interact with height and perceived dominance. These findings are consistent with results from recent studies in which gender has not been found to interact with the use of cues, such as postural openness, that affect perceived status [35]. The use of standardized photographs showing single actors in the present study may have reduced gender's influence by tempering obvious differences between our male and female targets' appearances. In addition, our studies were not designed or analyzed to specifically assess the influence of gender on perceived status, but to assess covariation in perceived size and dominance across targets who vary in age, gender, and appearance.

These data contribute to accumulating evidence that some nonverbal cues in humans and other animals may evolve their particular appearances in order to “piggyback” on perceivers' existing responses to certain stimuli. A common example is the use of low-frequency vocalizations during status competitions or aggressive encounters [44]. Because larger animals can produce lower frequency sounds, an animal that produces a lower-pitched sound may create the impression of larger size. Similarly, the specific appearances of some human facial cues may have evolved to generate the impression of physical maturity or immaturity [45], [46]. Angry expressions, for example, may help expressers achieve social goals by mimicking the appearance of morphological maturity and masculinity by simulating the low brows, small eyes, protuberant brow ridge, and thin lips of an adult male [46], [47]. Conversely, fearful expressions may mimic the appearance of an infantile face to elicit the attributions and behaviors that actual infants elicit from adults [46]. The present studies suggest that humans may also be able to use nonverbal status cues that simulate the appearance of body size to capitalize on pre-existing response tendencies to those appearance cues.

### Conclusions

In conclusion, these studies demonstrate that status cues, like postural openness, that humans use to convey social dominance create the appearance of changes in physical size, thereby shaping attributions of status. The results demonstrate convergence between human behaviors and the status displays of non-human animals and highlight the importance of low-level perceptual processes in shaping some of the complex processes that underlie human social behavior.
